# Assessment and Prediction of Response to Neoadjuvant Chemotherapy in Breast Cancer: A Comparison of Imaging Modalities and Future Perspectives

**DOI:** 10.3390/cancers13143521

**Published:** 2021-07-14

**Authors:** Valeria Romeo, Giuseppe Accardo, Teresa Perillo, Luca Basso, Nunzia Garbino, Emanuele Nicolai, Simone Maurea, Marco Salvatore

**Affiliations:** 1 Department of Advanced Biomedical Sciences, University of Naples “Federico II”, 80131 Naples, Italy; tperillo3@gmail.com (T.P.); maurea@unina.it (S.M.); 2 Department of Breast Surgery, Centro di Riferimento Oncologico della Basilicata (IRCCS-CROB), Rionero in Vulture, 85028 Potenza, Italy; gaccardo1987@gmail.com; 3 IRCCS SDN, 80143 Naples, Italy; luca.basso@synlab.it (L.B.); nunzia.garbino@synlab.it (N.G.); emanuele.nicolai@synlab.it (E.N.); marcosalvatore2.segreteria@gmail.com (M.S.)

**Keywords:** breast cancer, neoadjuvant chemotherapy, imaging, nuclear medicine, machine learning, artificial intelligence, radiomics

## Abstract

**Simple Summary:**

Nowadays patients affected by locally advanced breast cancer and particular subtypes of early breast cancer may benefit from neoadjuvant chemotherapy (NAC) before surgery with different advantageous, like reduction of tumor size and prognosis improvement. Pathological complete response to NAC is very variable amongst all different histological and immunophenotypic subtypes of breast cancer and its correct assessment by imaging is crucial for treatment planning, as patients could be addressed to conservative or demolitive breast surgery with reconstruction. Advanced imaging techniques, such as MRI and nuclear medicine, recently contributed to the prediction of chemotherapy response in the early phase of NAC, to avoid side effects and psychological implications of the oncological treatment in patients who are supposed to be unresponsive. This review article aims to compare different imaging techniques for both assessment and prediction of response to NAC and explain the new revolutionary contribute offered by Artifical Intelligence in this field.

**Abstract:**

Neoadjuvant chemotherapy (NAC) is becoming the standard of care for locally advanced breast cancer, aiming to reduce tumor size before surgery. Unfortunately, less than 30% of patients generally achieve a pathological complete response and approximately 5% of patients show disease progression while receiving NAC. Accurate assessment of the response to NAC is crucial for subsequent surgical planning. Furthermore, early prediction of tumor response could avoid patients being overtreated with useless chemotherapy sections, which are not free from side effects and psychological implications. In this review, we first analyze and compare the accuracy of conventional and advanced imaging techniques as well as discuss the application of artificial intelligence tools in the assessment of tumor response after NAC. Thereafter, the role of advanced imaging techniques, such as MRI, nuclear medicine, and new hybrid PET/MRI imaging in the prediction of the response to NAC is described in the second part of the review. Finally, future perspectives in NAC response prediction, represented by AI applications, are discussed.

## 1. Introduction

Neoadjuvant chemotherapy (NAC) is becoming the standard of care for locally advanced breast cancer [1] aiming to reduce tumor size before surgery. Indeed, NAC offers different advantages. It could reduce breast tumor size and/or downstage the axilla, allowing a more conservative surgery, and enables in vivo evaluation of treatment effectiveness, which allows changing the therapeutic strategy according to personal patient response. NAC was also found to be a valuable prognostic factor for patients who obtain a pathological complete response (pCR) as they seem to improve their survival [2]. Unfortunately, less than 30% of patients generally achieve pCR [3,4,5,6] and approximately 5% of patients show disease progression while receiving NAC [7]. Moreover, the therapeutic effect remains unknown until the patient has received at least two cycles of chemotherapy. In patients who are non-responders, treatment may be changed to a more effective regimen and avoid side effects from an ineffective treatment that can be discontinued. Early evaluation of tumor response could also allow earlier timing of surgery if the tumor appears refractory to NAC.

Thus, early prediction of the response to NAC could be an important weapon for individualized medicine. A prediction model could avoid patients to be overtreated with useless chemotherapy cycles which are not free from side effects and psychological implications. It is likewise crucial to accurately assess the response to treatment after NAC in order to establish the most appropriate surgical approach.

Imaging plays a fundamental role in the evaluation of patient candidates to NAC for the evaluation of primary tumor and axillary lymph-node status. Indeed, the main aim of imaging, particularly by means of ultrasound (US), digital mammography (DM)/digital breast tomosynthesis (DBT), is the assessment of the response to treatment and the estimate of residual tumor, both influencing patients’ prognosis and surgical strategy [8]. The introduction of advanced imaging techniques able to provide functional information and quantitative parameters reflecting tumor biology, such as magnetic resonance imaging (MRI) [9], contrast-enhanced spectral mammography (CESM), integrated positron emission tomography (PET) with computed tomography (CT) (PET/CT), as well as the newest hybrid PET/MRI scanner, may have a significant impact in the evaluation of patient candidates to NAC, not only improving the accuracy in detecting residual tumor, but also early predicting the response to treatment. Similarly, the application of artificial intelligence (AI) techniques may enable to early identify patients who may benefit from NAC, extracting quantitative meaningful parameters from baseline examinations.

The aim of this review is to elucidate and compare the role of conventional and advanced imaging techniques in assessing and predicting the response to NAC as well as to illustrate future perspectives represented by AI applications. A comprehensive summary of the major imaging techniques employed for the assessment and prediction of response to treatment is reported in Table 1.

## 2. Evaluation of Residual Tumor after NAC

Patients who undergo NAC are essentially monitored by a clinical breast examination (CBE), mammography (DM), ultrasound (US), and/or magnetic resonance imaging (MRI). The accurate assessment of breast tumor and regional lymph node response to preoperative systemic therapy is crucial and the selection of imaging methods prior to surgery should be determined by the multidisciplinary team, according the recent NCCN guidelines [10]. All these procedures are basically engaged to monitor changes of tumor size which is considered the main parameter to define the tumor responsivity to NAC, thus influencing the subsequent surgical decision. Chapgar et al. [8] first estimated the error of CBE, DM, and US in the detection of size of residual tumor after NAC; they found a significant correlation between the radiological and/or clinical size and the pathological one, but the level of correlation was only moderate for all the three procedures. Therefore, the authors invited surgeons to keep caution in the interpretation of residual tumor size in the preoperative time when using CBE, US, or DM. During the last decade, MRI and new technologies like DBT and Automated Breast Ultrasound (ABUS) have enforced the correlation between the radiological detection of residual tumor size after NAC and the pathological one, offering new perspectives in clinical practice.

### 2.1. Conventional Imaging Techniques

#### 2.1.1. Digital Mammography and Digital Breast Tomosynthesis

**Figure 1:**
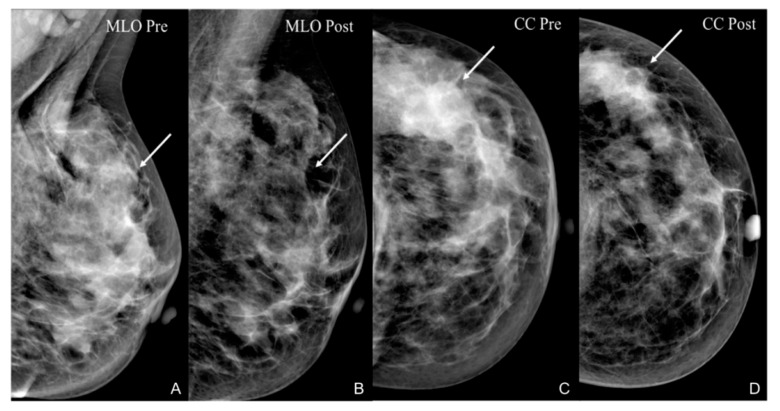
A 36-year-old patient with G3, Luminal B, HER2+ left breast cancer undergoing NAC. An irregular, hyperdense opacity with inner microcalcifications, determining distortion of the surrounding parenchyma, is depicted on both MLO (arrow in (**A**)) and CC (arrow in (**C**)) views. After NAC, the irregular opacity is no longer appreciable on both MLO (arrow in (**B**)) and CC, while some microcalcifications are still detectable as expression of in situ carcinoma (arrow in (**D**)) as revealed by histological specimen (pCR).

The accuracy of DM-based post-treatment evaluation depends upon specific tumor morphological characteristics. In particular, it is higher for lesions who appear well-circumscribed on the pre-treatment examination. The most reliable indicators of treatment response are decrease in size and density, whereas calcifications and spicules are the biggest challenge for imaging interpretation. Indeed, several authors correlated mammographic microcalcification with surgical specimen and concluded that residual microcalcifications on DM could be due to both residual tumor (i.e., in situ carcinoma) (Figure 1) and treated cancer with necrotic material, thus it did not always represent the residual tumor [11].

Kim et al. [12] demonstrated that the extent of microcalcifications on mammography after NAC did not correlate with the extent of residual cancer in 38.5% of the 96 women included in their single center retrospective study. Um et al. [13] supported the same conclusion and their single center retrospective study enhances how post-NAC residual microcalcifications on DM have a lower correlation with residual tumor size compared to MRI. Adrada et al. [14] reported that the residual calcified size and pathologic results differed by up to 22% in breast cancer patients undergoing NAC. Spicules as well are not an effective indicator of residual disease as they can possibly represent tumor itself after NAC or underlying fibrosis and hyalinization, as supported by Winchcombe et al. [15]. 

DBT has proven to be superior to DM for the evaluation of tumors size, especially in case of small lesions and dense breast [16]. Moreover, two studies have shown higher accuracy of DBT than DM in assessing response to NAC, though studies with bigger population are required to validate such results [17,18]. However, some limitations of DM and DBT are reported, related to a possible overestimation in residual tumor size measured by DBT, whereas underestimation when measured by DM [11]. Different factors could explain these findings, such as the superiority of DBT than DM in assessment of tumor margin and size, and also the detection of benign calcifications of necrotic tissue after treatment and/or the inclusion of fibrotic spicules in the measurement.

#### 2.1.2. Ultrasound 

US is considered a more accurate method than CE or DM in assessing tumor size and in the monitoring of residual breast tumors, but operator dependency and shortage of qualified personnel have been an issue in hand-held US [19]. Conflicting results have been described in literature about the utility of US in the evaluation of residual tumor after NAC. In a recent study, Dobruch-Sobczaka et al. [20] assessed the variability of breast tumor echogenicity from hypo to isoechoic after three or four courses of NAC. They found persistent tumor hypo-echogenicity after three courses of NAC to be predictive of a poor response to treatment. Indeed, the change in tumor echogenicity could predict a pathological response with significant accuracy and may be considered in NAC monitoring. Moreover, Evans and colleagues showed that a decrease in tumor stiffness at US sono-elastography, could be considered a good predictor of a pathological response already after the second cycle of NAC [21]. On the other hand, Baumgartner et al. [22] concluded that US imaging is insufficient to predict pCR with adequate accuracy with an overall sensitivity of 60.8% and specificity 78.0% for US predicted remission. In the same series, a multivariable analysis assessed the influence of receptor status on the diagnostic precision of US and pathologic outcome, thus supporting the results of previous series [23,24,25]. In particular, triple negative tumors seem to have the highest NPV and lowest FNR amongst all the receptor subtypes, implying that pathologic response to NACT can most reliably be predicted for this subtype.

In a recent retrospective study by Makanjuola et al. [26], US has shown a high predictive value of pCR when combined with mammography images. According to the authors, the complete radiological response (rCR) could be defined with the absence of a mass and normal breast parenchyma overlying the post-biopsy tissue marker both in mammography than ultrasound images. Indeed, according to this strict criteria, rCR evaluated both by DM and US correlated highly (approx. 93%) with the pCR in the histological exam after surgery, even if a longer follow up of patients and more prospective studies are mandatory to validate this innovative result.

New technologies have been introduced in breast US imaging such as the automated breast ultrasound (ABUS) which provides 3D images using wider probes. Shin et al. [27] enhanced how ABUS permits more appropriate image evaluation for architectural distortion and large breast mass compared to conventional breast US. However, in the series described by Park et al. [11], ABUS showed the lowest reliability in prediction of residual tumor size and pCR compared to DM, DBT, and MRI, as it tends to underestimate residual tumor. The authors also suggest that ABUS may be sensitive enough to distinguish chemotherapy-induced fibrosis and hypoechoic tumor after NAC. Furthermore, quantitative ultrasound (QUS) technique is a new imaging modality which analyzes raw, ultrasonic radio-frequency echoes (RF), thus defining quantitative parameters characteristic of the tissue. Unlike B-mode ultrasound (BUS), which defines anatomical information, QUS conveys tissue microstructure characteristics, including cell nuclei, by quantitatively analyzing the radiofrequency (RF) data backscattered from tissues [28,29]. Ultrasonic RF backscattered signals are basically analyzed by two types of QUS: the spectrum analysis of single-frame RF signals and RF time-series. Ultrasonic spectrum analysis of single-frame RF data has shown good results in tissue characterization when diagnosing prostate cancer, ocular tumors, and cardiac abnormalities, as well as detecting the early response to radiotherapy and chemotherapy based on detecting tumor microstructure changes and cell death in tumor according recent preclincal and clinical studies [30]. Oelze et al. used QUS parameters to characterize the difference between benign from malignant lesions in rodent models of breast cancer [31]; in their model, they also demonstrated that QUS could be also useful to recognize breast cancer micrometastasis in excised lymph nodes. In another study, QUS data were used to classify lesions as benign or malignant from 78 patients breast with a resulting sensitivity and specificity of 96% and 84%, respectively [32]. Studies have also demonstrated that time changes in QUS parameters reflect cell death [33], suggesting new frontiers for QUS parameters and textural analyses in the prediction and the monitor response to NAC in patients with LABC [34] at an earlier stage than the standard US imaging [35].

### 2.2. Advanced Imaging Techniques

NAC monitoring methods such as DM or US or physical examination are not free from limitations [19]. Sometimes, NAC-induced architectural changes can mask a reduction in primary tumor at imaging, even if a response is found at pathological examination.

For instance, Taxanes determine a decrease of enhancement of the tumor and the whole breast tissue as it has a specific anti-angiogenetic effect [36]. Functional imaging techniques, such as positron emission tomography (PET), MRI with diffusion weighted imaging, and diffuse optical spectroscopy, enable to capture changes in the microstructure, vascularization, and metabolic activity of tumors under the influence of chemotherapy after the first cycle of treatment. 

#### 2.2.1. Magnetic Resonance Imaging

MRI is currently used to provide an accurate assessment of primary lesion dimension, which is usually underestimated by DM and US, loco-regional disease spread, multifocality, multicentricity, and lymph nodes involvement. In particular, dynamic contrast-enhanced MRI (DCE-MRI) and diffusion-weighted MR imaging (DWI-MRI) significantly improved the detection, diagnosis, and monitoring of breast tumors using high-field (1.5–3.0 Tesla) scanners and dedicated radiofrequency coils. Of note, DCE-MRI is well known to enable the most accurate assessment of lesion response after NAC reflecting tumor tissue changes on the basis of contrast distribution (**37**), while DWI-MRI allows the calculation of apparent diffusion coefficients (ADC)—a quantitative measure of the diffusivity of water—providing information related to tumor cellularity and the integrity of cell membranes, being sensitive to intra-tumoral changes induced by chemotherapy. MRI has been proved to accurately evaluate residual tumor after NAC with high sensitivity (76–92%), specificity (60–89%), and accuracy (76–90%) [37]. Moreover, MRI is more accurate than DM, DBT, and US in evaluating residual tumor after NAC and predicting pCR [11], but its accuracy is not adequate to replace the pathological evaluation of breast tumor and axillary nodes, as recently confirmed by Weber et al. [38]. Overall, a metanalysis published by Wu et al. [39] in 2012 confirmed that DWI-MRI has a high sensitivity (0.93 with 95% CI 0.82–0.97) while CE-MRI (contrast enhanced) shows a high specificity (0.91 with 95% CI 0.87–0.94) in assessing and predicting a pathological response to NAC in breast cancer patients. The combined use of DW-MRI and CE-MRI has the potential to improve the diagnostic performance in monitoring NAC, but authors conclude that further large prospective studies are warranted to assess the actual value of this combination in breast cancer preoperative treatment screening. Hahn et al. retrospectively analyzed 78 breast MRI acquired after NAC and depicted that MRI diagnostic accuracy increases when DWI is associated to DCE-MRI (specificity 80%; accuracy 91%) [40]. More recently, a retrospective study by Choi et al. [41] concluded that the in-breast residual cancer burden index, which is an absolute assessment of residual tumor in the breast parenchyma and lymph nodes, correlated best with changes in DCE-MRI features, and the MRI-measured angio-volume reduction rate correlated best with pathologic tumor responses.

However, some concerns have been raised on the measurements of breast tumor lesions on MRI, mainly related to the different enhancement patterns of tumor lesions over time [42]. Furthermore, a great variability of MRI accuracy of residual tumor evaluation after NAC according to different histological subgroups is reported, as confirmed by Pasquero et al. [43], who showed in a short series the superiority of MRI accuracy in diagnosing HER2+ and triple negative tumors, but suggesting caution in case of luminal tumors’ evaluation. The relationship between the accuracy of MRI and subtype classification has been well described by Fukuda and al. [44]. The pCR rate of the triple negative subtype was higher than the luminal subtype. MRI for predicting pCR is generally more accurate in tumors that have a better response. It is also known that the ER-negative tumors have higher contrast uptake on MRI after NAC than the ER-positive ones. In the triple-negative subtype, the high contrast uptake may explain the high accuracy of MRI diagnosis.

In line with the latest results, Kim et al. [45] recently tried to identify MRI assessment criteria which could help clinicians to select appropriate patients for avoiding surgery after NAC when pCR is obtained. In particular, the authors found that lesion size and the lesion-to-background parenchymal signal enhancement ratio (SER) on early phase MRI images detected residual tumors with high sensitivity and NPV in Hormone Receptor (HR) negative (Triple negative and HER2+) breast cancers after NAC. Although these promising results, limitations of the study such as the lack of consideration of residual DCIS and axillary lymph node metastasis raise some controversies in the clinical practice, thus more prospective and enlarged studies are required to enforce the clinical value of the outcomes.

#### 2.2.2. Contrast-Enhanced Spectral Mammography 

CESM is a recent imaging technique that combines DM to intravenous administration of a contrast agent, thus allowing the assessment of neo-angiogenesis in patients who cannot undergo MRI [46]. In the study of Iotti et al., 46 patients were enrolled during and after NAC in order to compare residual tumor evaluation with CEM and MR. The two imaging techniques showed high agreement (0.76–0.96) though both of them underestimated residual tumor [47]. More recent but analogous results have been described by Patel et al. [48] in their short series where comparing CESM versus MRI for assessment of complete response, the sensitivity was 95% vs. 95%, specificity 66.7% vs. 68.9%, positive predictive value 55.9% vs. 57.6%, and negative predictive value 96.7% vs. 96.9%, respectively. Similarly, Barra et al. [49] concluded that CEM was comparable to MRI, showing that mean differences between CEM, MRI, and residual histopathological tumor size were 0.8 cm and 1.8 cm, respectively. Evidence from a recent metanalysis reports that CESM has equal pooled specificity (0.82) and greater sensitivity (0.83 vs. 0.77) compared to DCE-MRI [50].

### 2.3. Nuclear Medicine Techniques

Nuclear medicine offers valid experimental and clinical tools for the evaluation of tumor residual after NAC. While Kitajima et al. [51] reported that 18F−Fluorodeoxyglucose (FDG) positron emission tomography/computed tomography (PET/CT) showed a tendency toward underestimation of the residual tumor with relatively low specificity and PPV, an innovation of mammography imaging which uses ^18^F-FDG for the detection of breast cancer, positron emission mammography (PEM) or dedicated breast PET (dbPET) has shown higher sensitivity and specificity than PET/CT in particular for lesions smaller than 2.5 cm [46]. The prospective study of Noritake et al. compared PEM technique to whole-body (WB) ^18^F-FDG-PET and PEM proved to better detect residual tumor, whereas it was not superior in predicting pCR, though only 20 patients were enrolled [52]. In a series including 47 patients, Sasada et al. [53] concluded that PEM was more accurate than WB PET in detecting residual primary tumors after NAC, particularly intraductal carcinoma. Analogous results have been reported by the Japanese team of Koyasu [54] in 2018. Furthermore, recent advanced radiotracers other than 18F-FDG have been proposed to predict response to neoadjuvant therapy [55,56,57]. In particular, a biophysical mathematical model to predict tumor response for two HER2 + breast cancer patients was defined by Jarret et al. using quantitative data from MRI and ^64^Cu-DOTA-trastuzumab PET to estimate tumor density, perfusion, and distribution of HER2-targeted antibodies for each individual patient. In addition, Gong et al. proposed ^18^F-FES PET/CT, a non-invasive method to monitor estrogen receptor expression, to predict patient prognosis on the basis of changes in SUVmax tracer uptake in metastatic breast cancer. Furthermore, other novel radiotracers are being developed and applied for the in vivo measurement of different aspects of breast cancer, such as cell proliferation and tumor metastasis (^18^F-fluorothymidine), tissue hypoxia (^18^F-Fluoromisonidazole), receptor status, tumor antigen levels (^68^Ga-PSMA), and therapeutic response (^18^F-Fluciclovine) [57]. 

Molecular breast imaging (MBI), is a technique employing dedicated gamma cameras and an injected radiopharmaceutical such as technetium-99m Sestamibi, whose uptake is related to several biological issue, i.e., blood flow and mitochondrial activity. MBI is currently performed; (1) in breast cancer patients to assess disease extension, and response to neo-adjuvant chemotherapy, similarly to MRI; (2) for high-risk surveillance; and (3) in cases of equivocal mammographic/sonographic findings [58]. Hunt et al. [59] recently reported their experience in 90 patients comparing the accuracy of MRI and MBI in the detection of invasive breast cancer response to NAC. The authors concluded that MBI can be performed as an alternative in patients with contraindications for performing MRI, even if the pattern of response to NAC and alterations in tumor vascularity may affect the ability of MBI and MRI to detect residual disease. Notwithstanding, these promising techniques present some practical limitations as they are expensive and time-consuming, requiring intravenous injection of a radiotracer; thus, their use is now limited only in academic centers and mainly for research purposes. 

### 2.4. Artificial Intelligence

AI is emerging as a new paradigm in healthcare, allowing the possibility to use huge amount of data to make the prediction of interest, such as tumor characterization, response to a specific treatment, and patients’ prognosis using different algorithms. Radiomics is a method to extract quantitative parameters from medical images in order to obtain data that can be used to help diagnosis and make predictions using AI algorithms, e.g., machine learning and deep learning methods. Indeed, radiomics allows the extraction of quantitative features to depict information on pixel distribution within the image, reflecting its heterogeneity, that cannot be assessed by the human eye. Radiomics applications in breast tumor imaging are numerous and are continuously increasing, especially in the setting of prediction of response to NAC. Sutton et al. combined pre and post-NAC DCE-MRI images to assess delta radiomics features for classifying pCR [60]. They validated a combined radiomics and molecular subtype-based classifier model to predict pCR with high accuracy and reproducibility. As a result, the combination of both tumoral features outperformed single methods alone for detecting a pCR. Authors concluded that this model could improve radiologists’ performance, and also facilitate the standardization of post-NAC MRI reporting.

## 3. Prediction of Response to NAC

Tumor downsizing and pCR are the main aims of NAC. Its complete course usually requires months and the administration of different chemotherapy drugs. Therefore, early prediction of response is crucial for increasing survival, lowering toxicity and costs as it could avoid unnecessary further drug administration in patients who do not respond. In detail, advanced techniques such as MRI, hybrid-imaging and AI modalities have been proposed for this purpose.

### 3.1. Magnetic Resonance Imaging

**Figure 2:**
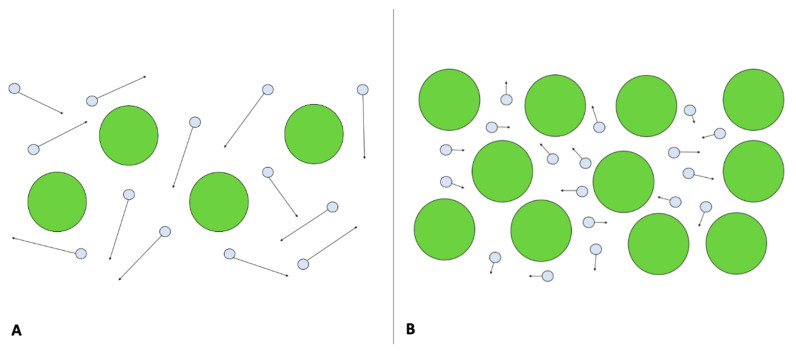
Diffusion weighed imaging. In (**A**), free diffusion of water molecules (circles with arrows) in the extracellular space, due to limited number of cells, is shown. In (**B**), an example of restricted diffusion of water molecules due to higher cellularity is shown.

**Figure 3:**
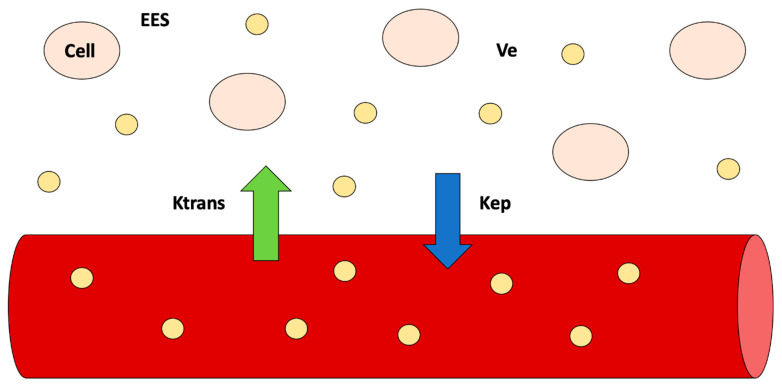
Dynamic contrast-enhanced MRI—significance of quantitative parameters according to the compartmental model. Quantitative parameters reflect exchanges of contrast agent concentration (yellow circles) between the plasma and extracellular extravascular space (EES) (Ktrans) and between EES and plasma (Kep). Ve represents the volume of EES.

The use of functional imaging techniques, such as DWI-MRI and DCE-MRI, aims at depicting biological properties of tumors, such as cellularity and neo-angiogenesis, trough the extraction of quantitative DWI-MRI (apparent diffusion coefficient, ADC) and DCE-MRI (Ktrans, Ve, Kep, iAUC) parameters that may change earlier during the course on NAC, before any morphological changes are detectable (Figure 2 and Figure 3).

**Figure 4:**
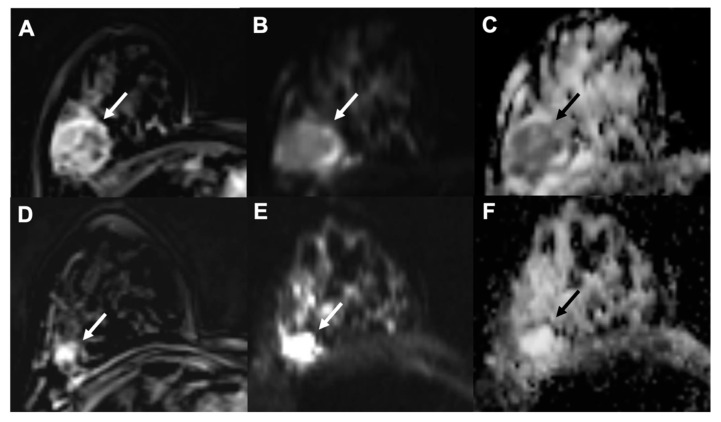
Example of early assessment of the response to NAC using diffusion weighted imaging (DWI). (**A**–**C**) = pre-NAC examinations; (**D**–**F**) = early assessment examination after two cycles of cytotoxic NAC. (**A**,**D**) = dynamic post-contrast images; (**B**,**E**) = DWI images; (**C**,**F**) = ADC maps. A 37-year-old patient with a G3, triple negative invasive ductal carcinoma of the right breast (white and black arrows). Early assessment showed a reduction of tumor size along with increase of signal intensity on ADC maps (**C**) compared to the pre-treatment examination (**F**). Pathology after surgical resection revealed pathological complete response (pCR).

In particular, Tourel et al. studied the role of DWI-MRI and ADC in this setting. Tumors with higher cellularity, and thus with lower ADC values, showed better response to NAC, whereas tumors with necrosis, which showed high ADC levels, were associated with worst survival outcome (Figure 4) [61]. 

**Figure 5:**
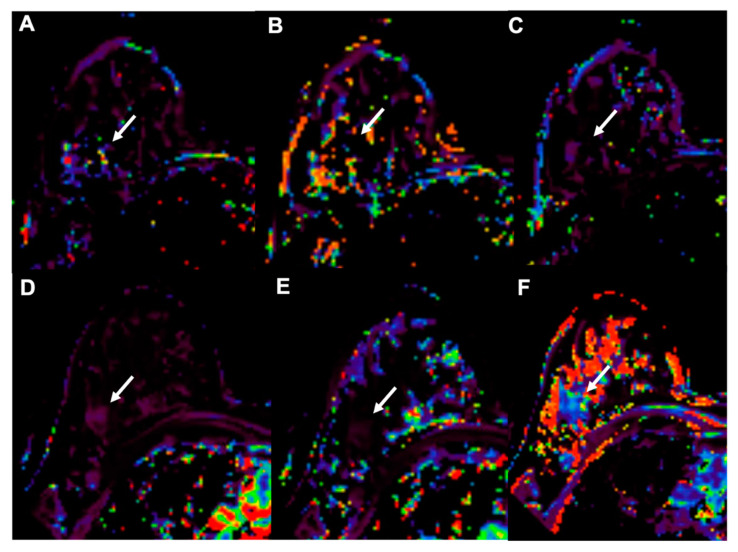
Example of early assessment of the response to NAC using dynamic contrast-enhanced imaging (DCE-MRI) in a 37-year-old patient with a G3, triple negative invasive ductal carcinoma of the right breast (arrows, same case shown in Figure 4). (**A**–**C**) = pre-NAC examinations; (**D**–**F**) = early assessment examination after two cycles of cytotoxic NAC. Ktrans (**A**,**D**), Kep (**B**,**E**) and Ve (**C**,**F**) maps. Early assessment showed a reduction of Ktrans (286 vs. 83.9 min^−1^) and kep (91.49 vs. 20.14 min^−1^ × 100) with a slight increase of Ve (275.34 vs. 308.08 × 1000) signal intensity on ADC maps (**C**) compared to the pre-treatment examination (**F**). Pathological complete response (pCR) was proved at pathology examination after surgical resection.

Although heterogeneous results regarding the use of DCE-MRI parameters are reported, it seems that the combination of quantitative perfusion parameters such as Ktrans, Kep, and Ve reflecting tumor permeability and cell density can early predict pCR during NAC (Figure 5) [62]. 

In this setting, Tudorica et al. [63] confirmed the same conclusion in their recent article. Their initial findings from a 28-patient cohort showed that changes in tumor neo-angiogenesis and permeability described byDCE-MRI quantitative parameters can be detected earlier than tumor size reduction after the first of six or eight cycles of NACT, supporting the hypothesis that functional changes precede morphological tumor variations at early NAC cycles. The authors found that the percent changes of the Ktrans, Ve, and Kep parameters, as well as the SSM-unique τi parameter, are good to excellent early predictors of pathologic response. Further studies are required to standardize and better understand the role of DWI and PWI as the values present in the literature are still heterogeneous. However, some methodological issues on DCE quantitative parameters measurements, significantly affecting their reliability, have been recently reported [64].

### 3.2. Hybrid Imaging Techniques

Hybrid-Imaging using PET/CT could be extremely useful in early response prediction as it assesses both morphological and functional cancer features. In particular, tumor cells change their metabolic expression after NAC, thus the assessment of glucose uptake rates might be of clinical value. Indeed, ^18^F-FDG PET/CT is routinely used for staging, recurrence evaluation, and treatment response. Whole-body ^18^F-FDG PET/CT enables metabolic activity assessment of breast tumors. Standardized uptake value (SUV) seems to correlate with histology as it is higher in high-grade invasive ductal carcinomas, triple negative, and erb-2 negative tumors and lower in low-grade lobular carcinoma. Furthermore, SUV is significatively lower in patients who experience pCR after the second cycle of chemotherapy [52]. Garcia Vicente et al. [65] also analyzed metabolic tumor features with ^18^F-FDG PET/CT. In their study including 67 patients, they found that volume-based metabolic variables obtained with ^18^F-FDG PET/CT such as metabolic tumor volume (MTV) and total lesion glycolysis (TLG), unlike SUV based variables (SUVmax, SUVmean, and SUVpeak), were good predictors of both NAC response and prognosis in locally advanced breast cancer. The main limitation of PET is the assessment of small tumors as it has low spatial resolution. PEM could overcome such limit as it is accurate for the evaluation of small lesions. In particular, Soldevilla et al. used PEM for interim prediction of the response to NAC in a retrospective study enrolling 108 patients. Though further studies are required to validate these results, SUVmax and lesion to background (LTB) showed strong correlation with pCR [66]. 

**Figure 6:**
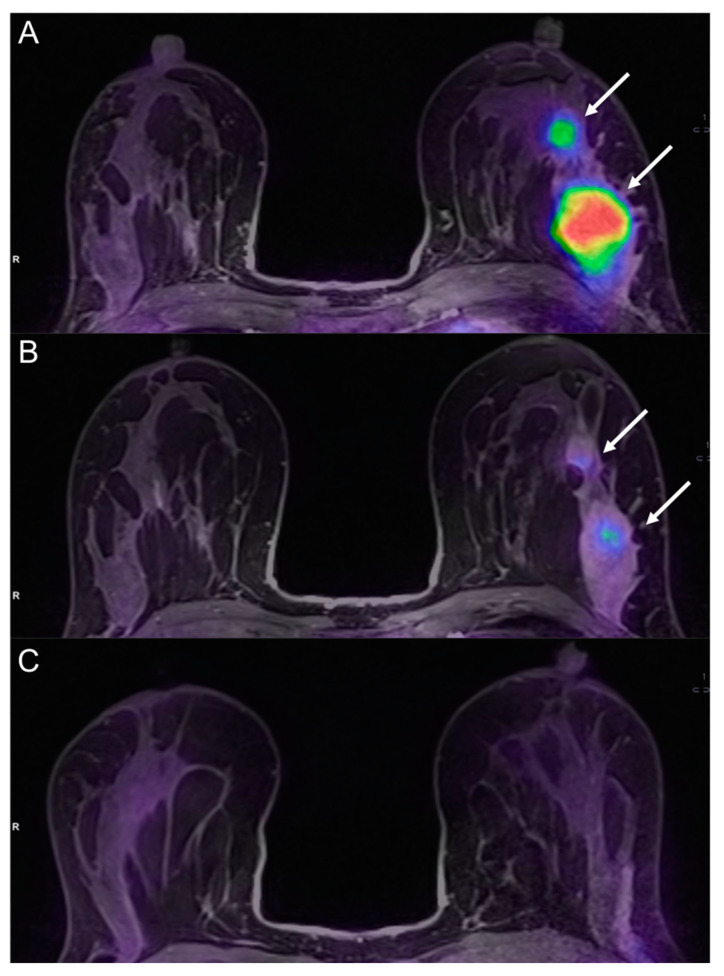
A 36-year-old patient with left breast cancer undergoing NAC (same patient shown in Figure 1). Fused PET/MRI images acquired before (**A**), during (**B**), and after (**C**) NAC are shown. While a slight reduction of the tumor and its satellite nodule (white arrows in **B**) is appreciable, ^18^F-FDG uptake is significantly reduced after the second cycle of chemotherapy (**B**) as compared to the pre-treatment evaluation (**A**). The tumor was not detectable at the post-treatment evaluation (**C**). Pathology after surgery demonstrated a complete response (pCR).

PET/MRI is the hybrid newest imaging technique which allows the simultaneous collection of morphologic, metabolic, and functional parameters with higher contrast resolution compared to PET/CT. It could be used for staging locally-advanced breast cancers and for monitoring and evaluating response to neoadjuvant and systemic chemotherapy [67]. A recent study by Cho et al. showed that ^18^F-FDG PET/MRI could be used to predict non-pCR after the first cycle of NAC. Specifically, the sensitivity significantly improved with the addition of MRI to PET parameters [68]. In a recent study by Wang et al. [69], 14 women with breast cancer were scanned with PET and MRI before and after the first or second cycle of treatment. The authors showed that percentage variations of SUVmax, TLG, and peak enhancement ratio (PER) were good predictors of NAC response (AUC 0.898, 0.878, and 0.837). In a study of 93 breast cancer patients, Pengel et al. [70] demonstrated that combination of PET/MR parameters in association with clinical data obtained the best accuracy in the detection of the response to NAC. In particular, age, breast cancer subtype, %change in SUVmax, and %change in largest tumor diameter on MRI were moderate predictors of pCR, while breast cancer subtype together with changes in SUVmax and tumor diameter provided the highest AUC (0.90). An et al. [71] also showed that combining data like DWI or DCE-MRI with PET improved negative predictive value and specificity values in comparison with the single examinations. An example of hybrid PET/MRI evaluation before, during, and after NAC is illustrated in Figure 6.

### 3.3. Artificial Intelligence

Different imaging methods have been explored in literature to detect early response to NAC using AI, including QUS, diffuse optical spectroscopy (DOS) [72], ^18^F-FDG-PET [73], but a great amount of literature focuses on MRI [74,75].

#### 3.3.1. Ultrasound

Differently from MRI and PET, which may have limitations in repeated use due to high costs and contrast agent/tracer injection, US is relatively inexpensive and safe for patient management. In particular, newest advanced QUS methods could be able to early depict changes in tumor microstructure without contrast agent administration. Fernandes et al. [76] used a machine learning model, such as Naïve Bayes classifiers, applied to strain elastography which was able to predict the response to NAC in locally advanced breast cancer as early as 2 weeks during treatment with high sensitivity (84%) and specificity (85%). These results may have a potential application in clinical practice. Moreover, the combination of quantitative data extracted from US elastography with histological data, such as Ki67 expression, was found to improve the predictive power [77]. Correlation between tumor stiffness and molecular subtype using shear-wave elastography was also investigated by Chang et al. [78]. In detail, 377 breast cancer lesions were evaluated using a multiple linear regression analysis which showed stiffness values were significantly influenced by tumor size, histological grade, and tumor subtype. Finally, Tadayyon et al. demonstrated in a large patient cohort [79] the hybrid QUS biomarkers including midband fit (MBF), spectral slope (SS), spacing among scatterers (SAS) could detect the response to NAC in locally advanced breast cancer lesions early after 4 weeks of therapy with relatively high sensitivity and specificity. This work confirmed the potential of QUS and machine learning methods for the early detection of breast tumor response, possibly helping clinicians to previously plan a personalized treatment for refractory patients. 

#### 3.3.2. Magnetic Resonance Imaging

Several studies analyzed the potential of AI with multiparametric MRI to predict response to early predict NAC. In this light, 38 patients underwent multiparametric MRI before and after two cycles of NAC in a study by Tahmassebi et al. with the extraction of both qualitative and quantitative parameters [80]. In detail, qualitative features were extracted from T2-weighted (e.g., signal intensity and presence of edema) and DCE images (e.g., tumor size, pattern of shrinkage, mass or non-mass enhancement, shape, margins, internal enhancement characteristics, distribution, and symmetry) while quantitative parameters were extracted from DCE (e.g., mean plasma flow, volume distribution, and mean transit time) and DWI images (e.g., minimum, maximum, and mean ADC values). Change in lesion size, complete pattern of shrinkage, mean transit time, peritumoral edema, and minimum ADC value resulted as the most significant variables for prediction of residual cancer. Some studies have set out how to predict response to NAC with only pretreatment imaging. For example, Cain et al. [81] demonstrated that multivariate machine learning-based models (e.g., SVM, LR) were able to accurately predict pCR, especially in TN/HER2þ + patient subgroup (*p* < 0.002) using pretreatment MRI performed in 288 patients. Braman et al. retrospectively evaluated 117 patients who underwent DCE-MRI and subsequent NAC. Combined intratumoral and peritumoral texture analysis-based radiomic approach was proved to predict pCR to NAC, independently of prior knowledge of receptor status suggesting their validity as response predictor [82]. Moreover, their findings suggest that the radiomic features most predictive of response vary across different receptor subtypes, and in particular, TN/HER2+ tumors were best characterized by a speckled enhancement pattern within the peritumoral region of non-responders. A direct consequence of this conclusion is that radiomics can also be helpful in identifying molecular subtypes of HER2+ from imaging thus helping treatment guidance [83]. Chamming’s et al. also used texture analysis-based radiomics to depict tumoral features associated to pCR from breast MRI acquired only before NAC [84]. According to their experience, kurtosis (a mathematical parameter which reflects tissue microstructure organization) seemed to be associated with pCR to NAC in non-triple negative breast cancer patients, and also represents a favorable biomarker for the identification of triple-negative breast cancer. Evidence from a multicenter study including 414 breast cancer patients who underwent NAC reported an accuracy of 0.86 obtained by a MRI-based radiomic signature, validated on an external dataset, for the prediction of pCR [85]. 

#### 3.3.3. Positron Emission Tomography

Radiomics applied to ^18^F-FDG PET offers the potential to non-invasively characterize tumor heterogeneity, a factor strongly related to tumor growth and resistance to medical treatments. However, reliable biomarkers have not been established yet. In this context, Li et al. recently published a retrospective study including 100 breast cancer patients who received NAC [86]. Radiomics predictors from pre-treatment ^18^F-FDG PET/CT scans were able to predict pCR after NAC especially when combined with patient age or Ki 67 levels from pre-treatment core needle biopsy specimens, as also demonstrated in the last prospective study by Luo et al. [87]. In their study, Li et al. also found a close association between radiomic features, receptor expression, and tumor T stage in line with previous studies, showing that the pCR rate varied with breast cancer molecular subtypes. Specifically, the TN and HER2-positive molecular subtypes showed a higher pCR rate after NAC. Antunovic et al. [88] supported this conclusion building different predictive models based on PET/CT radiomics, and finding an association between PET imaging features and pCR. The authors also found that patients with HER2+ and triple subtype were more likely to have a pCR to NAC than those with luminal subtype. Such evidence suggests that PET imaging features could be considered as potential predictors of pCR in locally advanced breast cancer patients.

## 4. Conclusions

Assessment of residual tumor and prediction of response in patients with locally advanced breast cancer undergoing NAC are clinically relevant to obtain the best outcome. Advanced imaging techniques are making headway in the assessment of such patients along with morphological and functional modalities. The possibility to extract information reflecting tumor biology from medical images could aid in the early identification of patients who will benefit from NAC, optimizing the response to treatment and avoiding unnecessary toxicity. In this perspective, hybrid imaging modalities and AI may have several and attractive future applications.

## Figures and Tables

**Table 1:** Summary of available imaging techniques for the assessment and prediction of response to neoadjuvant chemotherapy in breast cancer.

Assessment of Residual Tumor after NAC	
Morphological imaging techniques	Ultrasound—US
	Automated Breast Ultrasound—ABUS
	Quantitative ultrasound (QUS) methods
	Digital mammography—DM
	Digital breast tomosynthesis—DBT
Advanced imaging techniques	Contrast-enhanced spectral mammography—CESM
	Magnetic resonance imaging—MRI
	Dedicated breast positron emission tomography—DbPET
	Molecular Breast imaging—MBI
**Prediction of the Response to Treatment**	
Advanced imaging techniques	MRI diffusion weighted imaging—DWI
	MRI perfusion weighted imaging—PWI
	Hybrid imaging: PET/Computed Tomography (CT), PET/MRI
**Future Perspectives**	
Radiomics and Artificial Intelligence	Texture analysis, Machine Learning, Deep learning
